# Isolated Dipolar ONN Schiff Base Regioisomers: Synthesis, Characterization and Crystallographic Study

**DOI:** 10.3390/molecules29245863

**Published:** 2024-12-12

**Authors:** Pablo Castro-Tamay, David Villaman, Jean-René Hamon, Néstor Novoa

**Affiliations:** 1 Laboratorio de Química Inorgánica y Organometálica, Departamento de Química Analítica e Inorgánica, Facultad de Ciencias Químicas, Universidad de Concepción, Edmundo Larenas 129, Casilla 160-C, Concepción 4070386, Chile; pcastro@udec.cl (P.C.-T.); dvillaman@udec.cl (D.V.); 2 Univ Rennes, CNRS, ISCR (Institut des Sciences Chimiques de Rennes)—UMR 6226, F-35000 Rennes, France

**Keywords:** dipolar β-enaminone, ONN Schiff base, regioisomers, tautomeric form, X-ray diffraction

## Abstract

Organic compounds with 1,3-diketone or 3-amino enone functional groups are extremely important as they can be converted into a plethora of carbo- or heterocyclic derivatives or can be used as ligands in the formation of metal complexes. Here, we have achieved the preparation of a series of non-symmetrical β-ketoenamines (O,N,N proligand) of the type (4-MeOC_6_H_4_)C(=O)CH=C(R)NH(Q) obtained through the Schiff base condensation of 1,3-diketones (1-anisoylacetone, 1-anisyl-3-(4-cyanophenyl)-1,3-propanedione, and 1-anisyl-3-(4,4,4-trifluorotolyl)-1,3-propanedione) functionalized with electron donor and electron-withdrawing substituents and 8-aminoquinoline (R = CH_3_, 4-C_6_H_4_CN, 4-C_6_H_4_CF_3_; Q = C_9_H_7_N). Schiff base ketoimines with a pendant quinolyl moiety were isolated as single regioisomers in yields of 22–56% and characterized with FT-IR, ^1^H NMR, and UV-visible spectroscopy, as well as single-crystal X-ray crystallography, which allowed for the elucidation of the nature of the isolated regioisomers. The regioselectivity of the condensation of electronically unsymmetrical 1,3-diaryl-1,3-diketones with 8-aminoquinoline was studied by ^1^H NMR, providing regioisomer ratios of ~3:1 and ~2:1 in the case of CN and CF_3_ substituents, respectively. The electronic effects correlate well with the difference between the Hammett *σ*^+^ coefficients of the two *para* substituents on the aryl rings.

## 1. Introduction

**Chart 1:**
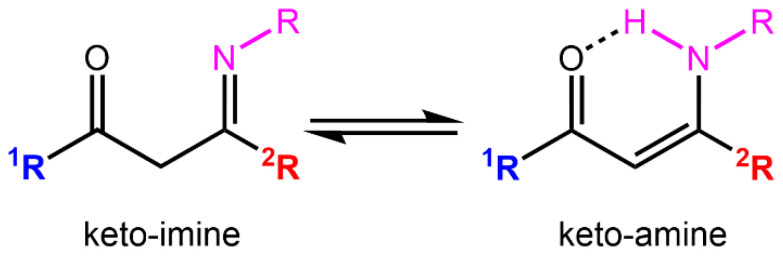
Tautomeric forms reported for ONN Schiff base derivatives obtained by condensation of β-diketones with primary amines; R^1^ = electron donor group and R^2^ = electron acceptor group.

Schiff bases, characterized by an imine (>C=N-) group [1,2], form an outstanding class of compounds that play a key role in organic chemistry and in the development of coordination chemistry due to their facile synthesis, structural flexibility, easily tunable electronic properties, versatility, and their ability to form stable complexes under various coordination geometries and oxidation states [3,4,5,6]. ONX-tridentate Schiff base proligands (X = O, N, S), resulting from the monocondensation of 1,3-diketones with primary amines [7,8,9], can exist in solution as a tautomeric mixture of ketoamine (enaminone, -(O=)C(^1^R)-CH=C(^2^R)-NH-) and ketoimine (iminone, -(O=)C(^1^R)-CH_2_-C(^2^R)=N-) forms (see Chart 1) [10].

When an unsymmetrical 1,3-diketone (R^1^ ≠ R^2^) is reacted with a primary amine, two regioisomers are possible, resulting from amine addition on the R^1^ or R^2^ side. The regioselectivity of the reaction has been demonstrated to be determined by a combination of steric and electronic factors; the condensation reaction at the carbonyl group adjacent to the less sterically hindered or more electron-withdrawing substituent is generally favored [11,12,13,14,15]. In their deprotonated forms, non-symmetrical bidentate β-enaminoketonato ligands are among the most ubiquitous chelating systems in coordination chemistry because they are easy to prepare and both their steric and electronic properties can be modified simply [16]. A lot of corresponding metal complexes bearing such pincer-like ligands containing nitrogen donors have, therefore, been prepared and have received increased attention as catalysts for organic synthesis [17,18] and for the preparation of L-lactide ring-opening polymerization initiators [12,13,14,19,20] and olefin polymerization catalysts [15]. β-Enaminoketonato metal complexes acting as metalloligands were also employed to construct new multimetallic architectures with desired properties [21,22,23].

On the other hand, it is worth mentioning that tridentate ONN Schiff base ligands derived from dipolar 1,3-diaryl-1,3-diketones functionalized with terminal electron donor (D) and electron acceptor (A) groups have not been reported. These new molecular systems are of great interest to our research field in pursuing the development of chelating chromophores with modulated push–pull properties for potential applications as functional molecular materials [24,25]. In this contribution, we report the successful synthesis, spectroscopic and structural characterization, and the regioselectivity study of a new class of tridentate ONN Schiff base proligands derived from appropriate 1,3-diaryl-1,3-diketones in which the aryl rings were electronically dissimilar but sterically identical by virtue of *para*-substitution, and bearing a planar quinolyl pendant donor.

## 2. Results and Discussion

### 2.1. Synthesis and Characterization

**Scheme 1:**
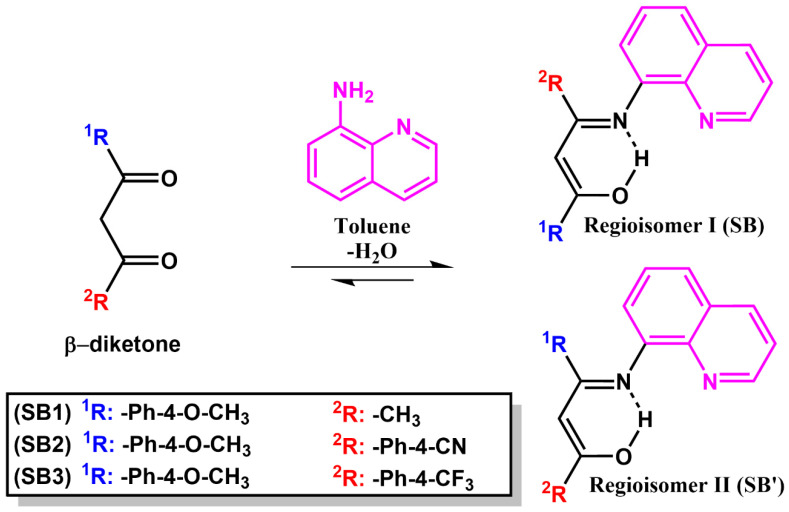
Synthetic pathway for obtaining ONN Schiff base molecules **SB1**–**SB3**. In **SB2** and **SB3**, both possible regioisomers I and II were detected in the bulk analysis.

Compounds **SB1**–**SB3** were synthetized through a Schiff base condensation reaction with 1,3-diketones (**β1**–**β3**), which were functionalized with electron donor and electron-withdrawing substituents with 8-aminoquinoline and were isolated as pure samples in yields of 22–56% upon column chromatography on silica gel, followed by fractional crystallization (see Section 3.3) (Scheme 1). The reactions were carried out in refluxing toluene, and the water generated was removed by azeotropic distillation. As expected, the condensation reaction was faster starting from the most electron-rich diketone, **β1**, being over after 16 h, while 24 h was needed to reach completion with the push–pull-type diketones **β2** and **β3** (TLC monitoring). The Schiff base derivatives **SB1**–**SB3** are air- and moisture-insensitive, thermally stable, and soluble in common organic solvents such as diethyl ether, DCM, chloroform, THF, and acetone, but they are sparingly soluble in n-hexane, methanol, and ethanol.

The Schiff base derivative **SB1** was formed as a single regioisomer, resulting from condensation taking place at the least impeded carbonyl functionality, which is the one nearest to the methyl group of the β-diketonic skeleton of **β1**. This follows the general trend observed for Schiff condensation of monosubstituted 1-R-1,3-butadione (R = organic or organometallic moieties) with primary amines [8,9,26,27,28]. The synthesis of compounds **SB2** and **SB3**, derived from Schiff condensation between the dipolar 1,3-diketones **β2** (MeO/CN) and **β3** (MeO/CF_3_) and 8-aminoquinoline, resulted in the formation of regioisomer mixtures **SB2/SB2′** and **SB3/SB3′**, respectively, due to the addition of 8-aminoquinoline to one or the other carbonyl group of the diketone (Scheme 1).

**Figure 1:**
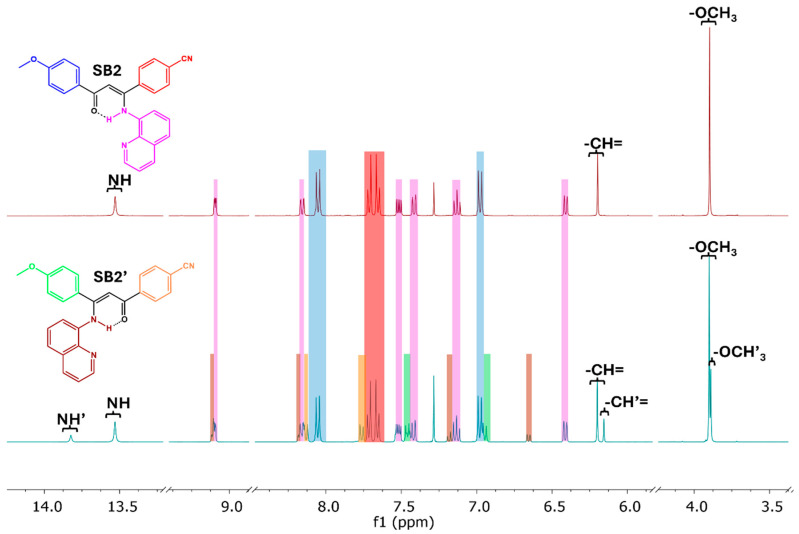
Comparative ^1^H NMR spectra of **SB2** (**top**) and regioisomer mixtures **SB2/SB2′** (**bottom**). Spectra acquired in CDCl_3_ at 400 MHz and 20 °C. The associated resonances are given in the color of the respective fragments in each case.

Analysis of the ^1^H NMR spectrum of each isomeric mixture, which gave rise to two sets of signals (see Figure 1 and Figures S13 and S14), allowed for the determination of the experimental yield of the condensation reactions and the relative formation ratio of the regioisomers. Comparison of the integration of the signals associated with the methine proton (δ = CH ~6.2 ppm, see Section 3.3 and Table S2) led to formation ratios of ~3:1 and ~2:1 for **SB2**:**SB2′** and **SB3/SB3′**, respectively. This correlates well with the electronic nature of the substituents. Indeed, according to Hammett’s coefficients (σ), the CN group (0.66), contained in the diketone precursor **β2**, has a higher electron-withdrawing ability than the CF_3_ substituent (0.54) borne by the diketone **β3** [29]. Such a behavior has previously been observed in the reaction of electronically unsymmetrical 1,3-diaryl-1,3-diketones with hydrazinopyridines to form 3,5-diarylpyrazolylpyridines [11]. The calculated experimental yields of the main reaction products for **SB2** and **SB3** were 46 and 49%, respectively.

The regioselectivity in the formation of enaminones is correlated with the electronic nature of the substituents at the *para* position. This resulted in the formation of a mixture of two regioisomers, with the largest amount of product formed by condensation of the carbonyl closest to the aromatic ring bearing the electron acceptor substituent. Related results have been reported for condensation reactions using 1,3-diaryl-1,3-diketones with terminal electron donor (D) and acceptor (A) substituents as precursors [11,30]. The relative ratio of the products formed increases with an increase in the electron donor/acceptor strength of the terminal substituents.

The formation of the regioisomers is very difficult to follow by thin-layer chromatography (TLC) and to separate by column chromatography. Alternatively, fractional crystallization using the fractions obtained from column chromatography (see Section 3.3) afforded single crystals of one of the two isomers, suitable for X-ray diffraction analysis (see Section 2.2), authenticating the presence of the two regioisomers in the bulk obtained by the Schiff base condensation reaction. Therefore, it was possible to isolate only one of the two isomers, which crystallizes preferentially to the other one, allowing the isolation of presumably the most abundant regioisomer in yields of 26 and 22% for **SB2** and **SB3**, respectively (see Section 3.3). The obtention of the single regioisomer compounds **SB1**–**SB3** was confirmed by FT-IR and ^1^H NMR spectroscopy. In addition, single-crystal X-ray diffraction analysis was performed to elucidate the molecular structure of the three Schiff base compounds (see Section 2.2).

The solid-state FT-IR spectra (KBr pellets) of the isolated regioisomers **SB1**–**SB3** (Figures S4–S6 and Table S1) exhibit the characteristic absorption bands of Schiff base compounds derived from 1,3-diketones reported in the literature [31]. The spectra show vibrational bands of strong and medium intensity between 1603 and 1545 cm^−1^, mainly associated with the stretching vibrations of C=O, C=N, and/or C=C. In addition, the spectrum of **SB2** displays a low-intensity band attributed to the stretching vibrations of C≡N at 2223 cm^−1^, while that of **SB3** shows a strong intensity absorption band associated to stretching vibration of C-F at 1322 cm^−1^ [32].

**Figure 2:**
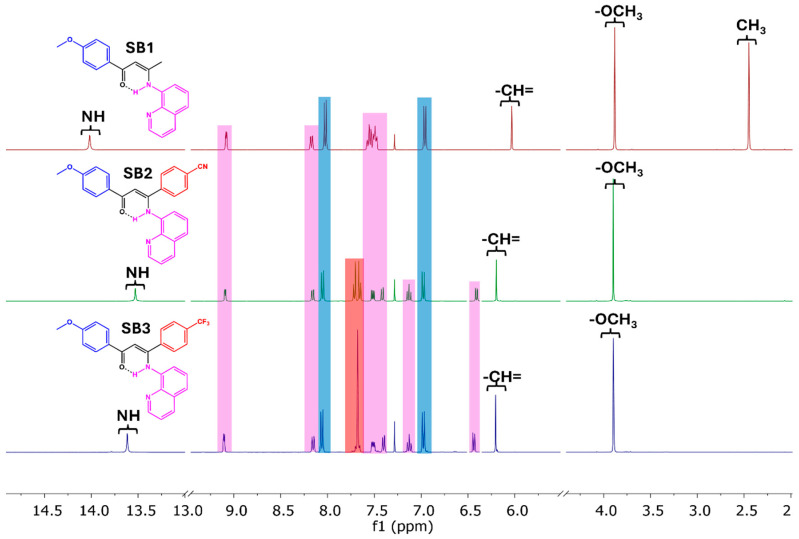
Comparative ^1^H NMR spectra of Schiff base compounds **SB1**–**SB3**. All spectra were acquired in CDCl_3_ at 400 MHz and 20 °C. The associated resonances are given in the color of the respective fragments in each case.

The exploration of the ^1^H NMR spectra of isolated compound **SB1**–**SB3** (Figure 2 and Figures S10–S12, Table S2) revealed signals associated with the keto-amine tautomer, showing three singlets between 3.87 and 3.88 ppm (3H), 6.03 and 6.21 (1H), and 13.53 and 14.01 (1H) ppm, attributed to the resonances of the methoxy (anisyl), methine (=CH-), and NH amine protons, in agreement with reported observations for a tautomeric enaminone structure [9,27]. The anisyl protons give rise to two doublets at 6.96–6.98 and 8.02–8.06 ppm, integrating both for 2H. On the other hand, in the **SB2** spectrum, the benzonitrile protons show up as two doublets at 7.66 and 7.71 ppm (2H:2H), while those of *p*-C_6_H_4_CF_3_ of **SB3** appear at a singlet at 7.68 ppm that integrates for 4H.

The resonances associated with the quinolinic fragment in **SB1** are seen as two doublet of doublets at 8.18 and 9.08 ppm, which integrate for 1H, and a multiplet at 7.57–7.47 ppm integrating for 4H. Those observations are in accordance with spectral data previously reported for Schiff base compounds derived from 1,3-diketones and 8-aminoquinoline [26]. On the other hand, the ^1^H NMR spectra of **SB2** and **SB3** exhibit the resonances associated with the heterocycle as a doublet at 6.41/6.44 ppm, a triplet at 7.13/7.13 ppm, and four doublets at 7.42/7.40 ppm, 7.52/7.51 ppm, 8.16/8.13 ppm, and 9.09/9.11 ppm, respectively; each of these resonances integrate for 1H. Although these chemical shifts slightly differ from those shown by **SB1**, they have also been reported for quinoline-derived Schiff base compounds [26]. The presence of an aromatic ring very close to the quinolinic residue in **SB2** and **SB3** would generate this change in the chemical displacements, with respect to **SB1**, associated with the magnetic anisotropy of the aromatic fragments. Comparable results were reported by Fritsch et al. [12,13,14,19,20] for the condensation of disubstituted 1,3-diketones with the same primary amine. The selectivity of the reaction was attributed to a steric factor rather than an electronic effect of the terminal substituents, resulting in the condensation of the quinolinic fragment with the carbonyl close to the less bulky group, such as methyl, *tert*-butyl, or even trifluoromethyl, when the other substituent was a phenyl fragment.

Finally, when comparing the ^1^H NMR spectra of the crude mixture of regioisomers with those of the isolated pure regioisomers **SB2** and **SB3** (Figures S11 and S12 vs. Figures S13 and S14, respectively), the presence of signals of lower intensity could be associated in both cases with the minor regioisomer formed. The singlet resonances for N-H show a low-field shift from 13.53 to 13.82 ppm and from 13.58 to 13.79 ppm, the resonance of the methine proton (=CH-) appears more shielded from 6.20 to 6.16 ppm and from 6.21 to 6.19 ppm, and the signal associated with the three protons of the methoxy group remains merely unchanged at 3.88 vs. 3.87 ppm. The spectra also revealed changes in the chemical shifts of the doublets of the aromatic rings. The doublet signals in **SB2**, at 8.05 and 6.98 ppm (*J* = 8.9 Hz) and at 7.71 and 7.66 ppm (*J* = 8.5 Hz), with each pair of doublets associated to an aromatic ring, are now observed at 7.46 and 6.95 ppm (*J* = 8.7 Hz) and at 8.13 and 7.76 ppm (*J* = 8.4 Hz), respectively. Similarly, for **SB3**, the doublets attributed to the protons of one aromatic ring observed at 8.06 and 6.98 ppm (*J* = 8.8 Hz) shifted to 7.48 and 6.95 ppm (*J* = 8.8 Hz). On the other hand, the singlet showing up at 7.65 ppm for the trifluoromethylated aromatic ring appears now as two doublets at 8.16 and 7.73 ppm (*J* = 8.4 Hz). These differences in the chemical shifts of the resonances attributed to the protons of the terminal aromatic rings are related to the change in the position of the quinolinic fragment when condensation occurs in the other carbonyl group, and the disturbance caused by the proximity of the aromatic fragments, as mentioned above.

### 2.2. Single-Crystal X-Ray Diffraction Analysis

**Figure 3:**
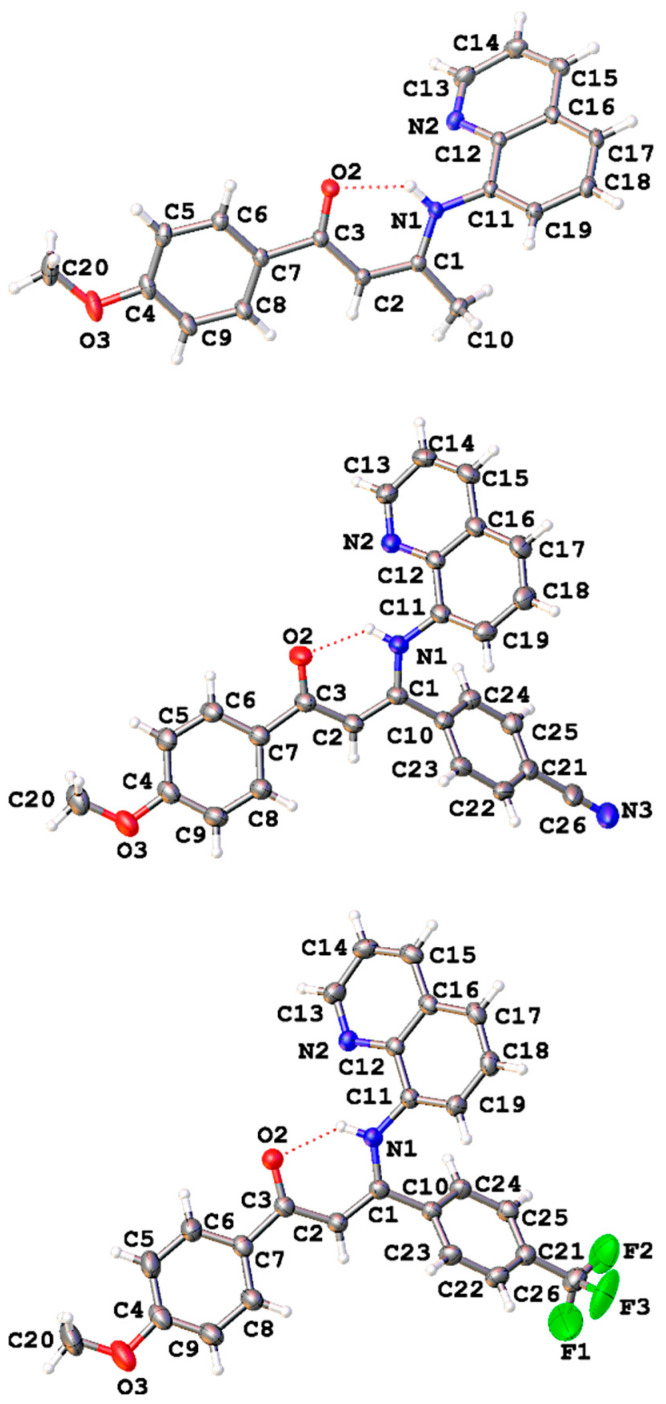
ORTEP views of Schiff base compounds **SB1** (**top**), **SB2** (**middle**), and **SB3** (**bottom**) with their respective atom numbering scheme. Thermal ellipsoids are drawn with 30% probability, and the intramolecular H-bond is plotted with a dashed line.

Diffraction-quality single crystals for X-ray structure investigation of the three Schiff base compounds **SB1**, **SB2**, and **SB3** were grown by slow evaporation of their respective saturated organic solution (see Section 3.3 for details). Single-crystal X-ray diffraction studies confirmed the proposed structure based on spectroscopic evidence (see above), exhibiting an ONN Schiff base architecture as the result of monocondensation of the 1,3-diketones (**β1**–**β3**) and 8-aminoquinoline. The molecular structures of **SB1**, **SB2**, and **SB3** are displayed in Figure 3 and Chart S1, with selected bond distances and angles gathered in Table 1. Compound **SB1** crystallizes in the monoclinic space group *P*2_1_*/n*, **SB2** crystallizes in the triclinic space group *P*$\bar{1}$, and **SB3** crystallizes in the monoclinic space group *P*2_1_*/c*, with a single molecule in the asymmetric unit in each case. The molecular structure confirms for each compound both the monomeric nature and that they adopt the *Z*-*s*-*Z* conformation, consistent with a keto-enamine tautomeric isomer [10]. Such enaminones may indeed have the following four isomers: *E*-*s*-*E*, *E*-*s*-*Z*, *Z*-*s*-*E*, and *Z*-*s*-*Z*, in which *E* and *Z* refer to two possible geometric conformations for the carbon–carbon double bond and *s*-*E* and *s*-*Z* refer to both isomerisms about the carbon–carbon single bond linking the anisoyl and the enamine fragment [10]. In the observed *Z*-*s*-*Z* conformational form, the mean plane (-O2-C3-C2-C1-N1H1-), the characteristic backbone of *β*-enaminone conformation, forms a pseudo six-membered aromatic ring. The C1-C2-C3 bond angles for **SB1**, **SB2**, and **SB3** are 124.35(9)°, 123.63(14)°, and 124.12(19)°, respectively. These values are very close to the idealized value of 120° for atomic systems with *sp*^2^ hybridization. Analysis of the O2-C3, C3-C2, C2-C1, and C1-N1 bond distances (Table 1) [33], with slight variations among the three compounds, indicates the presence of a conjugated *π*-electronic system through a pseudo-heterocycle formed as consequence of the intramolecular hydrogen bond N1-H1···O2 with d_N···O_ = 2.666(12) Å, 2.643(17) Å, and 2.645(2) Å, respectively, closing the planar pseudo six-membered ring through a resonant ···O2=C3-C2=C1-N1-H1 fragment [34]. All those metrical parameters compare favorably to previously reported structural data for variously substituted organic and organometallic enaminone derivatives [9,26,35].

In **SB1**–**SB3**, the aromatic ring (-C4-C5-C6-C7-C8-C9-) attached to the methoxy electron donor fragment forms dihedral angles with the central plane of 11.46°, 16.40°, and 13.33°, respectively. In addition, the second aromatic ring attached to the electron acceptor group (-C10-C24-C25-C21-C22-C23-), present in **SB2** and **SB3**, deviate by 50.96° and 49.18°, respectively, with respect to the enaminone mean plane. The pseudo heterocycle and the two planes contained in the quinolinic fragment, (-C11-C12-C16-C17-C18-C19-) and (-C12-N13-C14-C15-C16-), form dihedral angles with average values of 55.58(4)°, 42.32(6)°, and 36.01(7)° for **SB1**, **SB2**, and **SB3**, respectively.

**Figure 4:**
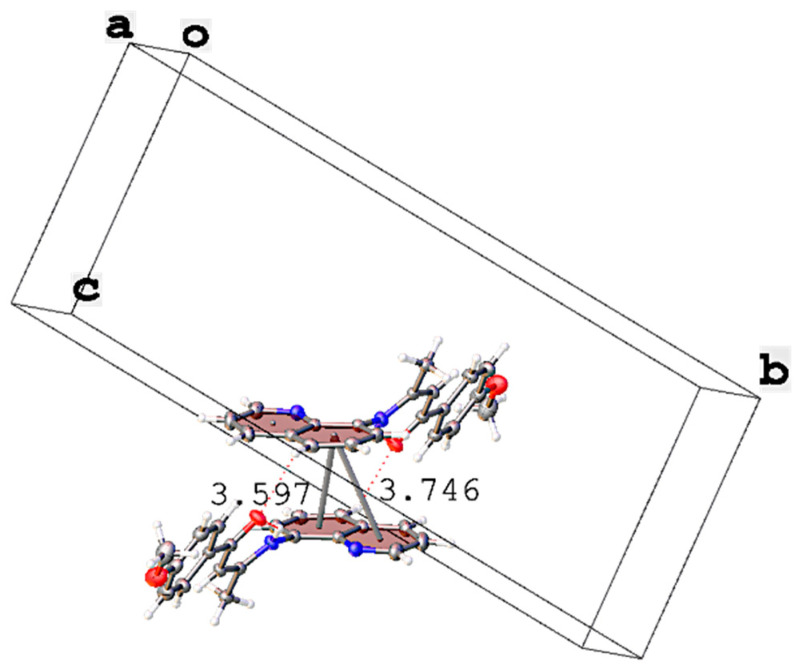
π–π antiparallel displaced interactions between (-C12-N13-C14-C15-C16-) and (-C11-C12-C16-C17-C18-C19-) with (-C11-C12-C16-C17-C18-C19-)^i^ in the crystal packing of **SB1**.

The stability of the crystal structure of compounds **SB1**–**SB3** was attributed to intermolecular hydrogen bond interactions and *π* interactions between the quinolinic fragments. The hydrogen bond distances and symmetry coordinates for intermolecular interactions are detailed in Table 2. For **SB1**, an antiparallel displaced π–π intermolecular interaction between aromatic ring planes in quinoline moieties was observed. Each aromatic ring in a quinolinic fragment represents a plane, (-C12-N13-C14-C15-C16-) and (-C11-C12-C16-C17-C18-C19-), with each plane interacting with another aromatic ring (-C11-C12-C16-C17-C18-C19-)^i^ of an adjacent quinoline moiety, with centroid–centroid distances of 3.597 and 3.746 Å and shift distances of 1.248 and 1.444 Å, respectively (Figure 4). In this conformation, the presence of weak intermolecular hydrogen interactions C17-H17···O2^i^ of 3.370 Å (donor–acceptor distance), and C19-H19···O2^ii^ of 3.480 Å, was observed.

**Figure 5:**
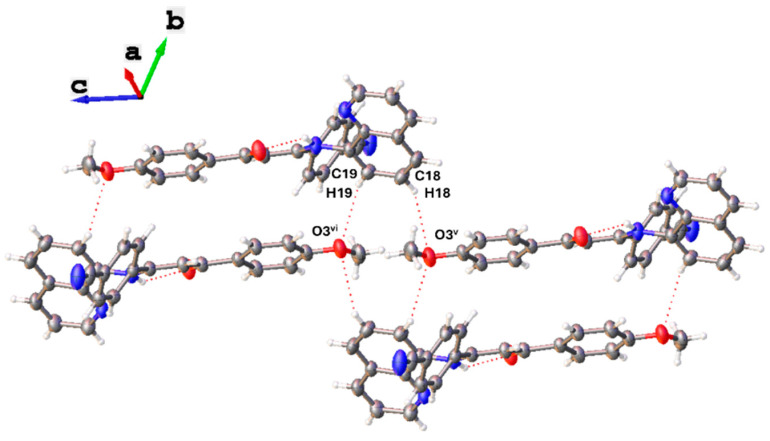
Intermolecular hydrogen bonds C18-H18···O3^v^ and C19-H19···O3^vi^ in the crystal packing of **SB2**.

The **SB2** crystal packing analysis reveals an antiparallel displaced overlapping between quinolinic moieties of two neighboring molecules, as described for **SB1**. In this conformation, the planes (-C11-C12-C16-C17-C18-C19-) and (-C12-N3-C14-C15-C16-) interact with the plane (-C12-N3-C14-C15-C16-)^iii^ of an adjacent molecule with centroid–centroid separations of 3.964 and 3.842 Å, respectively, and shift distances of 1.905 and 1.603 Å, respectively, values that are higher than the ones observed for **SB1**. The crystal structure of **SB2** is stabilized mainly by weak intermolecular hydrogen bond interactions C5-H5···O2^iv^ and C22-H22···O2^ii^ of 3.489 and 3.264 Å, respectively. These interactions occur between an oxygen atom in the *β*-diketoimine skeleton and aromatic hydrogens contained in phenyl-donor and phenyl-acceptor moieties, respectively. In addition, intermolecular hydrogen bond interactions C18-H18···O3^v^ and C19-H19···O3^vi^ of 3.496 and 3.353 Å between an oxygen atom in the anisyl fragment and aromatic hydrogens of two different quinoline fragments are also observed. As a consequence of these hydrogen interactions, an arrangement of stacked molecules with an alternating disposition of the electron donor and acceptor groups is observed (Figure 5).

**Figure 6:**
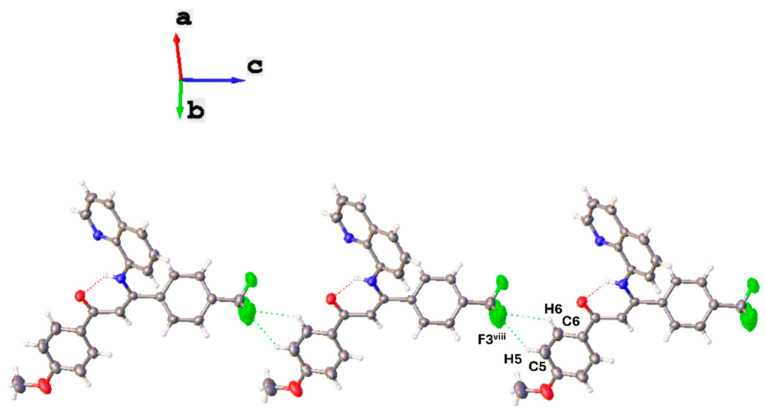
Intermolecular hydrogen bonds C6-H6···F3 ^viii^ and C5-H5···F3A ^viii^ in the crystal packing of **SB3**.

Lastly, the crystal structure of **SB3**, as described for **SB1**, is stabilized mainly by π–π intermolecular interactions between the quinolinic aromatic fragments; however, in this case, each aromatic ring plane presents only one π interaction. The interaction observed occurs between the planes (-C12-N3-C14-C15-C16-) and (-C11-C12-C16-C17-C18-C19-)^vii^ of quinoline moieties of adjacent molecules. Compared to what is observed for **SB1** and **SB2**, this interaction shows the shortest centroid–centroid and shift distances of 3.648 Å and 1.076 Å, respectively. Overlapping of the quinoline fragments of neighboring molecules, as observed for **SB1** (Figure 6), favors a weak intermolecular interaction C15-H15···O2^vii^ of 3.350 Å between the same molecules. In addition, intermolecular hydrogen bond interactions C6-H6···F3^viii^ and C5-H5···F3A^viii^ of 3.49 and 3.29 Å are observed. These interactions grow along the *c*-axis and result in a molecular arrangement with alternating donor and acceptor substituents (Figure 6). The packing is completed with C25-H25···O2^ix^ and C25-H25···N2^ix^ between hydrogen atoms in the aromatic ring bonded to the acceptor moiety and an oxygen atom in the main molecular skeleton (3.354 Å) and a nitrogen atom in the quinolinic aromatic moiety (3.370 Å). Finally, an intermolecular hydrogen bond C24-H24···F1A^x^ of 3.498 Å is observed (Figure S15).

### 2.3. Electronic Absorption Spectra of ***SB1**–**3***

**Figure 7:**
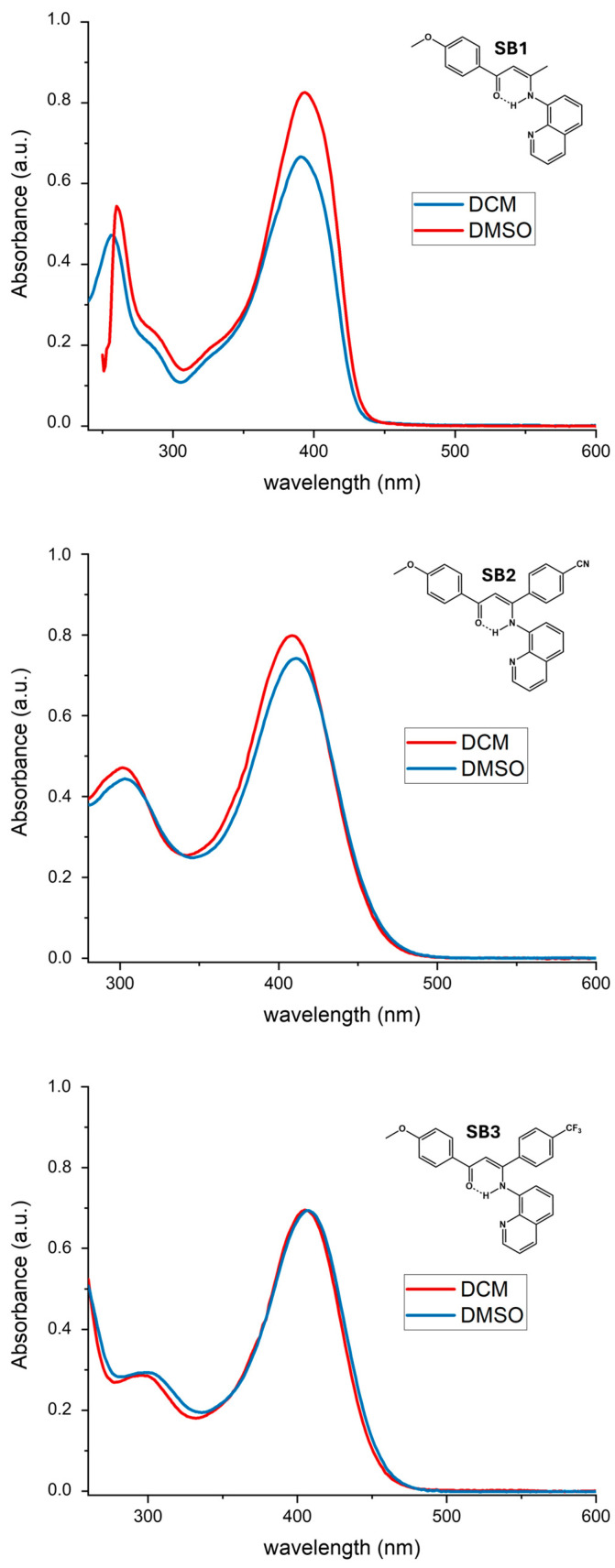
UV-vis spectra of the Schiff base compounds **SB1**–**SB3** (from **top** to **bottom**) recorded in dichloromethane (red line) and DMSO (blue line). Solutions at 20 °C.

The UV-visible absorption spectra of the three Schiff base compounds **SB1**–**SB3** were recorded in both dichloromethane (DCM) and dimethyl sulfoxide (DMSO) solutions at room temperature. The experimental spectra are presented in Figure 7 (see also Figure S16 for comparison with those of 1,3-diketone precursors **β1**–**3**), while their absorption maxima and log *ε* are collected in Table 3. They show three broad absorption bands between 250 and 253 nm, 294 and 304 nm, and 391 and 405 nm in DCM, in accordance with previous literature reports [26,36]. These bands are associated with π-π* charge transfer transitions and ligand-centered *n-*π* transitions due to the aromatic rings and the imine groups, thus belonging to ligand-to-ligand charge transfer (LLCT) transitions [37]. Switching to a higher polarity solvent (DMSO) induced a modest redshift (2–3 nm of the lowest energy band) in the spectra of compounds **SB1**–**SB3**. Thus, some solvatochromic behavior could be ruled out, a property desirable for photophysical applications such as nonlinear optics. However, this electronic absorption band shows a significant bathochromic change of 45–60 nm with respect to the corresponding *β*-diketone precursors (Figure S16). In this sense, they could be chelators of interest for the formation of coordination compounds with different applications in materials science [38].

## 3. Materials and Methods

### 3.1. Materials and Physical Measurements

Reactions were carried out under a dry nitrogen atmosphere using standard Schlenk techniques. Solvents were dried and distilled under dinitrogen by standard techniques [39]. 4-Methoxyacetophenone, potassium *tert*-butoxide, ethyl acetate, methyl 4-cyanobenzoate, sodium hydride, methyl 4-(trifluoromethyl)benzoate, *p*-toluene sulfonic acid, and 8-aminoquinoline were purchased from commercial suppliers and used without further purification. Column chromatography was carried out on silica gel 60 (70–230 mesh ASTM, Merck, Rahway, NJ, USA) as the stationary phase, using the eluents described in each synthetic procedure. Solid-state (KBr disk) FT-IR spectra were recorded on a Nicolet Magna IR-550 FTIR spectrometer in the 4000 to 400 cm^−1^ range. Absorption bands are expressed with $\tilde{\nu }$ in cm^−1^ with the following abbreviations to describe the intensity of the bands: vs = very strong, s = strong, m = medium, w = weak, and vw = very weak. Elemental analyses of **SB1**–**SB3** were conducted on a Fisons-EA-1108 CHNS-O Element Analyzer (Thermo Scientific, San Francisco, CA, USA) by the Renewable Resources Laboratory of the Faculty of Chemical Sciences, UdeC (Chile). The ^1^H NMR spectra were measured at 295 K with a Bruker Ascend-400 spectrometer (Bruker, San Jose, CA, USA). Chemical shifts (*δ*) are reported in ppm relative to tetramethylsilane (Me_4_Si). Coupling constants (*J*) are reported in Hertz (Hz), and the integrations are reported as the number of protons. The following abbreviations are used to describe peak patterns: s = singlet, d = doublet, dd = double doublet, t = triplet, and m = multiplet. UV-vis spectra were registered with a Merck Spectroquant Prove 600 spectrophotometer on compound solutions using quartz cuvettes with a 1.0 cm optical path. Analysis was performed quantitatively using dichloromethane (DCM) and dimethyl sulfoxide (DMSO) solutions at 3.0 × 10^−3^ mol·L^−1^.

### 3.2. Synthesis of β-Diketone (***β1***–***β3***)

CH_3_-O-C_4_H_4_-C(O)-CH_2_-C(O)-CH_3_ (**β1**): This compound was synthesized through a previously reported Claisen condensation reaction [40], using, in our case, a new procedure of purification. A Schlenk tube was charged with a magnetic stir bar, 5.00 g (33.29 mmol) of 4-methoxyacetophenone, 5.60 g (49.93 mmol) of potassium *tert*-butoxide, and 40 mL of THF. The mixture was stirred for 30 min at room temperature, giving a light yellow precipitate. Then, 5.00 mL of ethyl acetate and 20 mL of THF were added, and vigorous stirring was maintained for 16 h. Next, the solid was filtered off and washed with 3 × 50 mL of diethyl ether. The recovered solid was poured into 100 mL of deionized water and 30 mL of HCl 10% *v/v* and gently stirred. The solid was extracted with 3 × 50 mL of methylene chloride. The organic phases were collected and dried over anhydrous sodium sulphate, and the solvent was evaporated under reduced pressure. The resulting microcrystalline light pink solid was dried under vacuum for 5 h. Yield: 4.59 g (72%). FT-IR (KBr, cm^−1^): 3430 (w) ν(O-H), 3067, 3008 (vw) ν(=C-H aryl), 2971 (w), 2832 (w) ν(C-H alkyl), 1595 (s) ν(C=O), 1499 (s) ν(C=C aryl), 1261 (m), 1184 (m) ν(-C-O), 830 (m), 783 (m) g (C-H). ^1^H NMR (400 MHz, CDCl_3_) δ, keto-enol tautomer: 16.31 (s, 1H), 7.86 (d, *J* = 8.9 Hz, 2H), 6.94 (d, *J* = 8.9 Hz, 2H), 6.11 (s, 1H), 3.86 (s, 3H), 2.17 (s, 3H); diketo tautomer: 7.92 (d, *J* = 8.9 Hz, 2H), 6.94 (d, *J* = 8.9 Hz, 2H), 4.05 (s, 2H) 3.86 (s, 3H), 2.17 (s, 3H). These data are spectroscopically identical with known material [40].

CH_3_-O-C_4_H_4_-C(O)-CH_2_-C(O)-C_4_H_4_-CN (**β2**): The following procedure was adapted from a report of Duncan et al. [11]. A two-necked round-bottom flask was charged with a magnetic stir bar, 1.00 g (6.66 mmol) of 4-methoxyacetophenone, 1.39 g (8.66 mmol) of methyl 4-cyanobenzoate, 394 mg (9.99 mmol) of NaH, and 15 mL of THF. The mixture was refluxed for 16 h under a dinitrogen atmosphere. The resulting solution was cooled down to r.t., supplemented with 10 mL of deionized H_2_O and 5 mL of HCl 10% *v*/*v*, and vigorously stirred. The reaction mixture was extracted with DCM (3 × 20 mL), and the organic fractions were collected and dried over anhydrous sodium sulphate. The solvent was removed under reduced pressure, providing a red-orange solid. The crude product was recrystallized from hot ethanol to give a pure yellow solid. Yield: 1.39 g (75%). FT-IR (KBr, cm^−1^): 3411 (w) ν(O-H), 3071 (vw), 3024 (vw) ν(=C-H aryl), 2928 (w), 2827 (vw) ν(C-H alkyl), 2231 (m) ν(C≡N), 1595 (s) ν(C=O), 1504 (s) ν(C=C), 1265 (m) ν_asy_(-C-O-aryl), 1174(m) νs(-C-O-aryl), 835 (m), 788 (m) g(=C-H). ^1^H NMR (400 MHz, CDCl_3_) δ 16.84 (s, 1H), 8.08 (d, *J* = 8.4 Hz, 2H), 8.02 (d, *J* = 8.9 Hz, 2H), 7.80 (d, *J* = 8.4 Hz, 2H), 7.02 (d, *J* = 8.9 Hz, 2H), 6.82 (s, 1H), 3.93 (s, 3H).

CH_3_-O-C_4_H_4_-C(O)-CH_2_-C(O)-C_4_H_4_-CF_3_ (**β3**): This compound was synthesized using an analogous procedure to that previously described for **β2**, using 1.00 g (6.66 mmol) of 4-methoxyacetophenone, 1.39 g (1.39 mL, 8.66 mmol) of methyl 4-(trifluoromethyl)benzoate, and 394 mg (9.99 mmol) of NaH. Yield: 1.58 g (74%) of pale yellow solid. FT-IR (KBr, cm^−1^): 3428(w) ν(O-H), 3091 (vw), 3010 (vw) ν(=C-H aryl), 2919 (w), 2844 (w) ν(C-H alkyl), 1604 (s) ν(C=O), 1499 (s) ν(C=C aryl), 1327 (s) ν(C-F), 1251 (m) ν_asym_(-C-O-aryl), 1166 (s) νs(-C-O-aryl), 848 (m), 798 (m) g(=C-H). ^1^H NMR (400 MHz, CDCl_3_) δ 16.90 (s, OH, 1H), 8.09 (d, *J* = 8.1 Hz, 2H), 8.02 (d, *J* = 9.1 Hz, 2H), 7.76 (d, *J* = 8.2 Hz, 2H), 7.02 (d, *J* = 8.7 Hz, 2H), 6.83 (s, 1H), 3.92 (s, 3H). These spectroscopic data agree with the published ones [41].

### 3.3. Synthesis of Schiff Bases (***SB1***–***SB3***)

CH_3_-O-C_6_H_4_-C(O)-CH=C(CH_3_)-NH-(8-C_9_H_7_N) (**SB1**): This compound was synthetized through a Schiff base condensation reaction reported by Fritsch et al. [26]. A two-necked round-bottom flask was charged with 10 mL of toluene, 600 mg (3.12 mmol) of **β1,** and *p*-toluene sulfonic acid in a catalytic amount (~5.0 mg), and the mixture was stirred for 20 min under a dinitrogen atmosphere. Then, 359 mg (2.40 mmol) of 8-aminoquinoline was added, and the reaction was kept under reflux for 16 h using a system equipped with a Dean–Stark apparatus. The progress of the reaction was followed by silica-coated TLC plates using a mixture of hexane/ethyl acetate (2:1). The reaction mixture was allowed to cool down to room temperature, and the solvent was removed under vacuum. The solid residue was purified by column chromatography using a hexane/ethyl acetate (4:1, *v*:*v*) mixture as the eluent. The intense yellow band was collected, and the solvent was evaporated under reduced pressure to give a bright yellow solid. Yield: 435 mg (56%). Suitable single crystals for X-ray diffraction crystallography were grown by slow evaporation of a hexane/ethyl acetate (4:1) solution. Analysis calculated for C_20_H_19_N_2_O_2_ (319.38 g mol^−1^): C, 75.21; H, 6.00; N, 8.77. Found: C, 75.01; H, 6.24; N, 8.51. FT-IR (KBr, cm^−1^): 3437 (w) ν(N-H), 3066 (vw) ν(=C-H aryl), 2934 (w) ν_asym_(C-H), 2846 (vw) νs(-C-H), 1590 (s) ν(C=O), 1555 (s) ν(C=C aryl) and/or ν(C=N), 1225 (M) δ(C-O-aryl), 1164 (m) ν(C-O-aryl)., 1280 (m) ν(C-H), 1106 (m), 827 g(=C-H). ^1^H NMR (400 MHz, CDCl_3_) δ 14.01 (s, 1H), 9.08 (dd, *J* = 4.1, 1.7 Hz, 1H), 8.18 (dd, *J* = 8.3, 1.7 Hz, 1H), 8.02(d, *J* = 8.8, 2H), 7.52 (m, 4H), 6.96 (d, *J* = 8.9, 2H), 6.03 (s, 1H), 3.88 (s, 3H), 2.45 (s, 3H).

CH_3_-O-C_6_H_4_-C(O)-CH=C(C_6_H_4_-CN)-NH-(8-C_9_H_7_N) (**SB2**): This compound was synthesized using a similar procedure to that previously described for **SB1**, in this case using 630 mg (1.79 mmol) of **β2**, 284 mg (1.97 mmol) of 8-aminoquinoline, and a reaction time of 24 h. The obtained solid material was purified by column chromatography using a mixture of hexane/ethyl acetate (6:1) as the eluent. An intense yellow band was collected in several fractions (20 mL). The different solutions were allowed to evaporate at room temperature for 3 to 4 days to afford yellow needle-shaped crystals. The crystals were combined and washed with small amounts of diethyl ether followed by hexane. A crystal from this crop was selected for X-ray diffraction determination. Yield: 189 mg (26%). Analysis calculated for C_27_H_20_N_3_O_2_ (418.47 g mol^−1^): C, 77.49; H, 4.82; N, 10.04. Found: C, 76.97; H, 4.67; N, 10.31. FT-IR (KBr, cm^−1^): 3440 (w) ν(N-H), 3057 (vw) ν(=C-H aryl), 2927 (w) ν_asy_(C-H), 2848 (w) νs(C-H), 2227 (m) ν(C≡N), 1603 (s) ν(C=O), (w), 1545 (s) ν(C=C aryl) and/or ν(C=N), 1177 ν_asy_(-C-O-aryl), 1105 νs(-C-O-aryl), 800 γ (=C-H). ^1^H NMR (400 MHz, CDCl_3_) δ 13.53 (s, 1H), 9.09 (dd, *J* = 4.2,1.7 Hz, 1H), 8.16 (dd, *J* = 8.3, 1.7 Hz, 1H), 8.05 (d, *J* = 8.9 Hz, 2H), 7.71 (d, *J* = 8.6 Hz, 2H), 7.66 (d, *J* = 8.4 Hz, 2H), 7.52 (dd, *J* = 8.3, 4.2 Hz, 1H), 7.42 (dd, *J* = 8.3, 1.1 Hz, 1H), 7.13 (t, *J* = 7.9 Hz, 1H), 6.98 (d, *J* = 8.9 Hz, 2H), 6.41 (dd, *J* = 7.8, 1.2 Hz, 1H), 6.20 (s, 1H), 3.88 (s, 3H).

CH_3_-O-C_6_H_4_-C(O)-CH=C(C_6_H_4_-CF_3_)-NH-(8-C_9_H_7_N) (**SB3**): This compound was synthesized using a similar procedure to that previously described for **SB1**, using in this case 340 mg (1.05 mmol) of **β3**, 166.5 mg (1.15 mmol) of 8-aminoquinoline and 24 h of reaction. The solid residue was purified by column chromatography using a mixture of hexane/THF (4:1) as the eluent. An intense yellow band was collected in several fractions (20 mL). The different fractions were allowed to evaporate at room temperature for 3 to 4 days to obtain yellow square-sheet-like crystals. The crystals were combined and washed with small amounts of cold diethyl ether followed by hexane. A crystal from this crop was selected for X-ray crystallography. Yield: 103 mg (22%). Analysis calculated for C_26_H_20_N_2_O_2_F_3_ (449.44 g mol^−1^): C, 69.48; H, 4.49; N, 6.23. Found: C, 69.22; H, 4.12; N, 6.51. FT-IR (KBr, cm^−1^): 3431 (w) ν(N-H), 3077 (vw) ν(=C-H aryl), 2948 (m) ν_asy_(-C-H), 2834 (w) νs(C-H), 1563 (s) ν(C=O), (w), 1545 (s) ν(C=C aryl) and/or ν(C=N), 1322 (s) ν(C-F), 1225 ν_asy_(-C-O-aryl), 1173 νs(-C-O-aryl), 847 g(=C-H), 726 (w). ^1^H NMR (400 MHz, CDCl_3_) δ 13.58 (s, 1H), 9.11 (dd, *J* = 4.3, 1.7 Hz, 1H), 8.13 (dd, *J* = 8.3 Hz, 1.7 1H), 8.06 (d, *J* = 8.8 Hz, 2H), 7.68 (s, 4H), 7.51 (dd, *J* = 8.4, 4.1 Hz, 1H), 7.40 (dd, *J* = 8.2, 1.2 Hz, 1H), 7.13 (t, *J* = 8.0 Hz, 1H), 6.98 (d, *J* = 8.9 Hz, 2H), 6.44 (dd, *J* = 7.7, 1.3 Hz, 1H), 6.21 (s, 1H), 3.87 (s, 3H).

### 3.4. Single-Crystal X-Ray Structure Determinations

Molecular structures were determined using the single-crystal X-ray diffraction technique. Intensity data were collected in a BRUKER D8 VENTURE dual-source Cu/Mo system using Mo-Kα radiation (λ = 0.71073 Å) at 296 K. Intensity data were processed with the APEX4 package and were corrected for absorptions using SADABS [42]. Structures were solved and refined with OLEX2 1.5 [43] by least squares with ShelXL [44] and ShelXT [45] using the intrinsic phasing method. The crystallographic and refinement data for compounds **SB1**–**SB3** are given in Table 4. All non-hydrogen atoms were refined with anisotropic thermal parameters. The hydrogen atoms were positioned and refined with riding coordinates, except for the methyl terminal groups which were treated as a rotating group. The disorder of the -CF_3_ fragments in **SB3** was fixed with rigid body restraints (RIGU) with a standard deviation (sigma) of 0.004 for 1–2 and 1–3 distances. Additionally, to restrain the distances equivalently, the same distance (SADI) with a sigma of 0.02 was applied for C-F distances. The crystallographic cif files have been deposited within the Cambridge Structural Database with the identifiers CCDC 2390921-2390923, containing the supplementary crystallographic data for this paper. These data can be obtained free of charge via www.ccdc.cam.ac.uk/data_request/cif, accessed on 2 December 2024; by emailing data_request@ccdc.cam.ac.uk; or by contacting The Cambridge Crystallographic Data Centre, 12 Union Road, Cambridge, CB2 1EZ, UK.

## 4. Conclusions

In summary, we have reported the synthesis of a series of non-symmetrical β-1-anisyl-ketoen-3-amines bearing a pendant quinolyl donor through Schiff base condensation of readily available 1,3-diketones containing varying degrees of electron-donating and electron-withdrawing substituents and 8-aminoquinoline. The three new enaminones were characterized spectroscopically and crystallographically, including an examination of the role of sterics and electronics in the observed regioisomer ratios. The regioselectivity of the condensation of electronically unsymmetrical 1,3-diaryl-1,3-diketones with 8-aminoquinoline was studied by ^1^H NMR, providing regioisomer ratios of ~3:1 and ~2:1 in the case of CN and CF_3_ substituents, respectively. The electronic effects correlate well with the difference between the Hammett *σ*+ coefficients of the two *para* substituents on the aryl rings. The regioisomers were separated upon column chromatography and fractional crystallization, and the structure of the isolated ones was established with single-crystal X-ray diffraction analysis, showing that the Schiff condensation reactions took place at the carbonyl group adjacent to the electron-deficient substituent. ONN-tridentate Schiff base ligands are known to act as anionic pincer-type ligands to efficiently chelate metal ions to generate versatile, conformationally rigid metalloligands, thus allowing the construction of new multimetallic architectures with desired properties and applications, such as second-order nonlinear optics, sensors, molecular magnets, catalysts, and many others [3,22,46]. We believe the push–pull enaminones reported here also have promise for coordinating metal centers to build new molecular materials, and such experiments are in progress.

## Figures and Tables

**Table 1:** Selected bond distances (Å) and angles (°) of **SB1**–**SB3**.

**Bond Distance (Å)**	**SB1**	**SB2**	**SB3**
**C1-C10**	1.4976 (14)	1.484 (2)	1.491 (3)
**N1-C1**	1.3418 (12)	1.3563 (18)	1.351 (3)
**C1-C2**	1.3839 (13)	1.370 (2)	1.369 (3)
**C2-C3**	1.4251 (14)	1.436 (2)	1.438 (3)
**O2-C3**	1.2547 (12)	1.2480 (19)	1.250 (3)
**C3-C7**	1.499 (14)	1.488 (2)	1.483 (3)
**O3-C4**	1.3687 (15)	1.3703 (19)	1.393 (3)
**N3-C26**		1.140 (2)	
F1-C26 *			1.3173 (7)
**C21-C26**		1.438 (2)	1.464 (3)
**Bond Angle (°)**	**SB1**	**SB2**	**SB3**
**O2-C3-C7**	117.86 (9)	118.29 (14)	118.65 (19)
**O2-C3-C2**	123.04 (9)	121.96 (14)	121.60 (19)
**C1-C2-C3**	124.35 (9)	123.63 (14)	124.12 (19)
**N1-C1-C2**	120.60 (9)	121.79 (14)	121.38 (19)
**C2-C1-C10**	119.36 (9)	119.15 (13)	118.77 (18)
**N1-C1-C10**	119.98 (9)	118.81 (12)	119.62 (17)
**C1-N1-C11**	128.36 (9)	129.12 (14)	130.64 (18)

* Average distance.

**Table 2:** Hydrogen bond distances and symmetry coordinates for crystal structures of **SB1**–**SB3**.

Compound	D-H···A	d(D-H)/Å	d(H-A)/Å	d(D-A)/Å	D-H-A/°
**SB1**	N1-H1···O2	0.86	1.97	2.666 (12)	136.8
	C17H17···O2 ^i^	0.93	2.61	3.370 (13)	139.3
	C19H19···O2 ^ii^	0.93	2.83	3.480 (16)	128.4
**SB2**	N1-H1···O2	0.86	2.03	2.643 (17)	127.7
	C5H5···O2 ^iv^	0.93	2.73	3.489 (2)	139.4
	C22-H22···O2 ^ii^	0.93	2.48	3.264 (2)	142.7
	C18-H18···O3 ^v^	0.93	2.92	3.496 (3)	121.8
	C19-H19···O3 ^vi^	0.93	2.73	3.353 (2)	125.3
**SB3**	N1-H1···O2	0.86	1.98	2.645 (2)	133.7
	C15-H15···O2 ^vii^	0.93	2.76	3.350 (3)	122.4
	C6-H6···F3 ^viii^	0.93	2.91	3.490 (8)	121.4
	C5-H5···F3A ^viii^	0.93	2.61	3.293 (9)	130.6
	C25-H25···O2 ^ix^	0.93	2.70	3.354 (3)	128.3
	C25-H25···N2 ^ix^	0.93	2.63	3.370 (3)	136.8
	C24-H24···F1A ^x^	0.93	2.85	3.498 (7)	127.9
**Symmetry coordinates**					
^i^ 1 − x, 1 − y, 2 − z	^ii^ −1 + x, +y, +z	^iii^ 1 − x, 1 − y, −z		^iv^ 2 − x, −y, 1 − z	
^v^ −1 + x, +y, −1 + z		^vi^ 1−x, −y, 1−z		^vii^ 1 − x, 1 − y, 1 − z	
^viii^ +x, +y, −1 + z		^ix^ +x, 1/2 − y, 1/2 + z		^x^ +x, 1/2 − y, −1/2 + z	

**Table 3:** UV-vis absorption data for the Schiff base compounds **SB1–SB3**.

Compound	Band	*λ*/nm (log ε) CH_2_Cl_2_	*λ*/nm (log ε) DMSO	Solv. Shift (cm^−1^)
**SB1**	A	257 (4.67)	260 (4.73)	3
	B	287 (4.26)	289 (4.35)	2
	C	391 (4.82)	393 (4.91)	2
**SB2**	A	253 (5.01)	260 (4.86)	7
	B	301 (4.67)	303 (4.64)	2
	C	408 (4.90)	411 (4.87)	3
**SB3**	A	251 (4.84)	260 (4.70)	9
	B	294 (4.45)	293 (4.46)	−1
	C	405 (4.84)	407 (4.84)	2

**Table 4:** Crystal data collection and structure refinement for compounds **SB1**–**SB3**.

Compound	SB1	SB2	SB3
Empirical formula	C_20_H_18_N_2_O_2_	C_26_H_19_N_3_O_2_	C_26_H_19_F_3_N_2_O_2_
Formula mass, g mol^−1^	318.36	405.44	448.43
Temperature, K	296.15	296.15	296.15
Crystal system	monoclinic	triclinic	monoclinic
Space group	P2_1_/n	P$\bar{1}$	P2_1_/c
a (Å)	7.4516 (3)	8.1445 (3)	17.6152 (6)
b (Å)	22.3305 (10)	10.5294 (3)	9.2876 (3)
c (Å)	10.1970 (4)	12.8440 (4)	13.5040 (4)
α (°)	90	98.0710 (10)	90
β (°)	109.8860 (10)	103.4230 (10)	92.3570 (10)
γ (°)	90	90.2180 (10)	90
Volume (Å3)	1595.58 (12)	1060.00 (6)	2207.42 (12)
Z	4	2	4
D_calc_ (g cm^−3^)	1.325	1.270	1.349
μ (mm^−1^)	0.087	0.082	0.103
F(000)	672.0	424.0	928.0
Crystal size (mm^3^)	0.25 × 0.22 × 0.16	0.29 × 0.24 × 0.069	0.26 × 0.26 × 0.11
2Θ range (°)	4.622–72.64	4.734–70.148	4.628–67.604
Range, hkl	−12/12, −37/37, −16/16	−13/13, −16/16, −20/20	−27/27, −14/14, −21/21
No. refl. collected	47,985	51,112	59,859
No. independent refl.	7732	9346	8847
Data/restraints/parameters	7732/0/219	9346/0/281	8847/93/327
Goodness-of-fit on F2	1.017	1.023	1.022
Final R indexes [I >= 2σ (I)]	R1 = 0.0555, wR2 = 0.1539	R_1_ = 0.0685, wR_2_ = 0.1955	R1 = 0.0817, wR2 = 0.2406
Final R indexes (all data)	R1 = 0.0908, wR2 = 0.1804	R_1_ = 0.1392, wR_2_ = 0.2434	R1 = 0.1385, wR2 = 0.2897
Largest diff. peak and hole (e Å^−3^)	0.39/−0.20	0.26/−0.16	0.66/−0.32

## Data Availability

The original contributions presented in this study are included in the article/Supplementary Material. Further inquiries can be directed to the corresponding authors.

## Supplementary Materials

The following supporting information can be downloaded at: https://www.mdpi.com/article/10.3390/molecules29245863/s1, Chart S1: Molecular structure with atomic numbering of **SB1**–**SB3**; Figures S1–S3: Solid-state FT-IR spectra of 1,3-diketone precursors; Figures S4–S6: Solid-state FT-IR spectra of compounds **SB1**–**SB3**; Figures S7–S9: ^1^H NMR spectra of 1,3-diketone precursors; Figures S10–S12: ^1^H NMR spectra of compounds **SB1**–**SB3**; Figure S13: ^1^H NMR spectra of regioisomeric mixture of compounds **SB2** and **SB2′**; Figure S14: ^1^H NMR spectra of regioisomeric mixture of compounds **SB3** and **SB3′**; Figure S15: Intermolecular hydrogen bonds in **SB3**; Figure S16: Overplot UV-visible spectra of 1,3-diketone precursors and their tridentate ONN **SB1**–**SB3**. Table S1: Most important FT-IR absorptions of 1,3-diketone precursors and **SB1**–**SB3**; Table S2: ^1^H NMR chemical shifts of 1,3-diketone precursors and **SB1**–**SB3**.
