# Supplementary material for: Isolated Dipolar ONN Schiff Base Regioisomers: Synthesis, Characterization and Crystallographic Study
Source: Molecules. 2024 Dec 12;29(24):5863. doi: 10.3390/molecules29245863 (PMC11678688; doi:10.3390/molecules29245863)
Supplement: Supplementary file 1 [file molecules-29-05863-s001.zip › molecules-3327538-supplementary.pdf]

Supplementary Materials

Isolated dipolar ONN-Schiff base Regioisomers: synthesis, characterization and crystallographic study.

Pablo Castro-Tamay[1], David Villaman[1], Jean-René Hamon [2,] and Néstor Novoa[1,]**

1 Laboratorio de Química Inorgánica y Organometálica, Departamento de Química Analítica e Inorgánica, Facultad de Ciencias Químicas, Universidad de Concepción, Edmundo Larenas 129, Casilla 160-C, Concepción, Chile.

2 Univ Rennes, CNRS, ISCR (Institut des Sciences Chimiques de Rennes) – UMR 6226, F-35000 Rennes, France.

** Corresponding authors.*
E-mail address: *jean-rene.hamon@univ-rennes1.fr (J.-R. Hamon)*
E-mail address: *nenovoa@udec.cl (N. Novoa)*

Contents Page

Table S1. FTIR most important absorptions of compounds **1- 3** and **SB1-SB3**. 2
Figures S1-S3. Solid-state FTIR spectra of compounds **1- 3**. 2
Figures S4-S6. Solid-state FTIR spectra of compounds **SB1-SB3**. 4
Chart S1. Molecular structure with atomic numbering of **SB1-SB3**. 5
Figures S7-S9. ^{1}H NMR spectra of compounds **1- 3**. 6
Figures S10-S12. ^{1}H NMR spectra of compounds **SB1-SB3**. 7
Table S2. ^{1}H NMR chemical shifts of compounds **1- 3** and **SB1- SB3**. 9
Figure S13. ^{1}H NMR of regioisomeric mixture of compounds **SB2** and **SB2’**. 9
Figure S14. ^{1}H NMR of regioisomeric mixture of compounds **SB3** and **SB3’**. 10
Figure S15. Intermolecular hydrogen bonds in **SB3**. 10

Figure S16. Overplot UV-visible spectra of precursor **1-3**, and their tridentate ONN **SB1-SB3** 11

Table S1. FTIR most important absorptions of compounds β1-β3 and **SB1-SB3**.[a]

	β1	β2	β3	**SB1**	**SB2**	**SB3**
ν(O-H)	3430 (w)	3411 (w)	3428 (w)	………….	………….	………….
ν(N-H)	………….	………….	………….	3437 (w)	3440 (w)	3431 (vw)
ν(=C-H aril)	3067 (vw) 3008 (vw)	3071 (vw) 3024 (w)	3091 (w) 3010 (vw)	3066 (vw)	3057 (vw)	3077 (w)
ν(C-H)	2971 (w) 2832 (w)	2928 (w) 2827 (vw)	2919 (w) 2844 (w)	2934 (w) 2846 (vw)	2927 (w) 2848 (w)	2948 (w) 2834 (vw)
ν(C≡N)	………….	2231 (w)	………….	………….	2227 (w)	………….
ν(C=O)	1595 (vs)	1595 (vs)	1604 (m)	1590 (s)	1603 (s)	1563 (vs)
ν(C=N)/ ν(C=C aril)	1499 (m)	1504 (m)	1499 (m)	1555 (s)	1545 (s)	1545 (s)
ν(C≡F)	………….	………….	1327 (vs)	………….	………….	1322 (vs)
ν^{as}(-C-O-aril)	1261 (m)	1265 (m)	1251 (m)	1225 (m)	1253 (S)	1225 (m)
ν^{s}(-C-O-aril),	1184 (m)	1174 (m)	1166 (s)	1164 (m)	1170 (s)	1173 (m)
γ (C-H)	830 (m) 783 (m)	835 (m) 788 (m)	848 (m) 798 (m)	827 (m)	806 (vs)	847 (m)

[a]Solid-state FTIR spectra recorded as KBr disk, wavelengths in cm^{-1}.

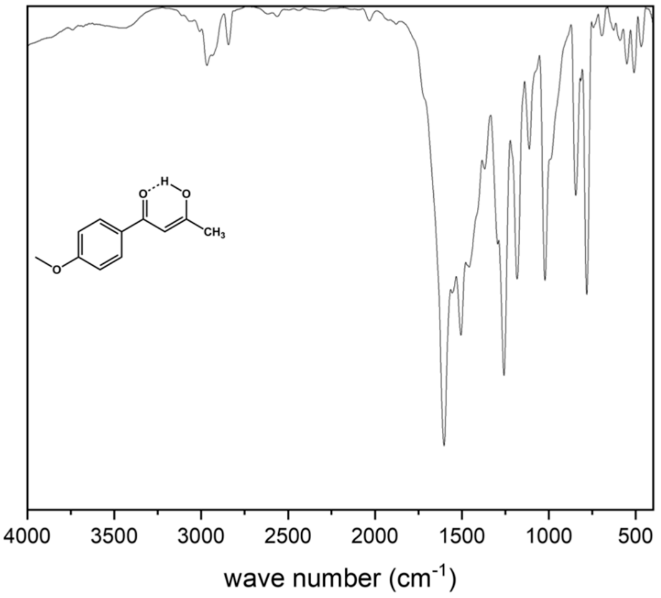

Figure S1. Solid-state FT-IR (KBr) of compound β1.

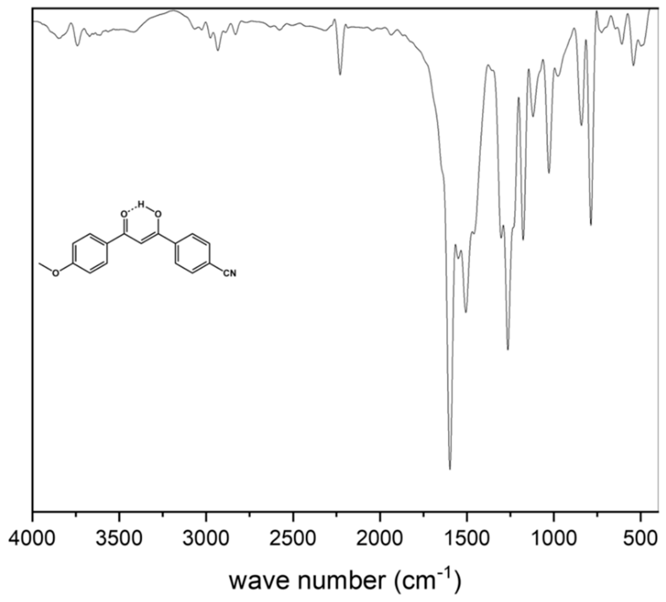

Figure S2. Solid-state FT-IR (KBr) of compound β2.

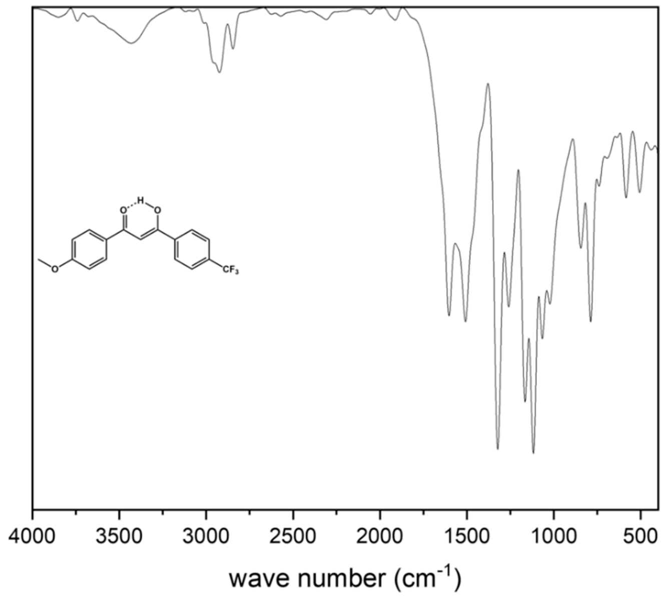

Figure S3. Solid-state FT-IR (KBr) of compound β**3**.

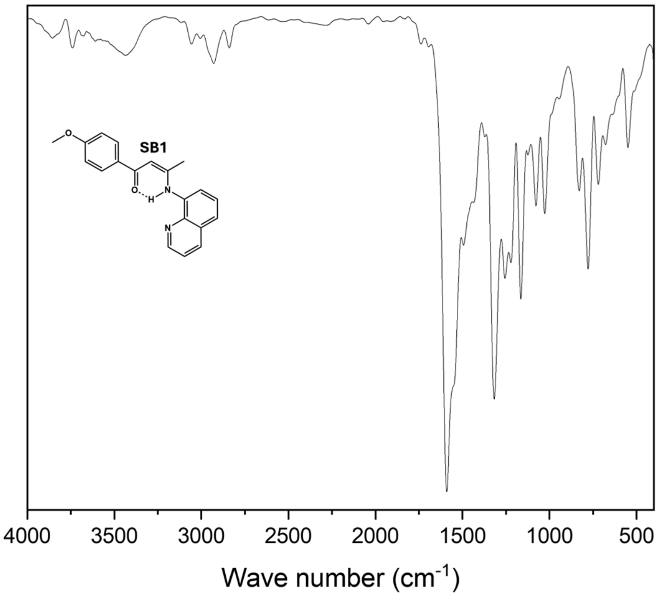

Figure S4. Solid-state FT-IR (KBr) of compound **SB1**.

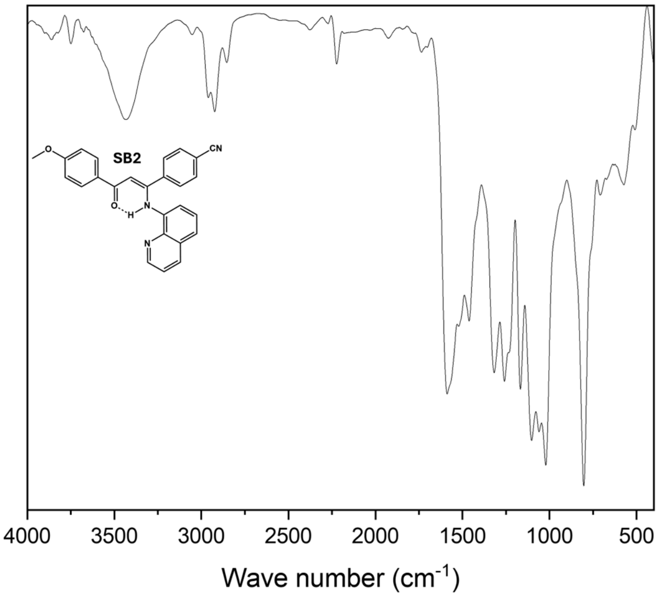

Figure S5. Solid-state FT-IR (KBr) of compound **SB2**.

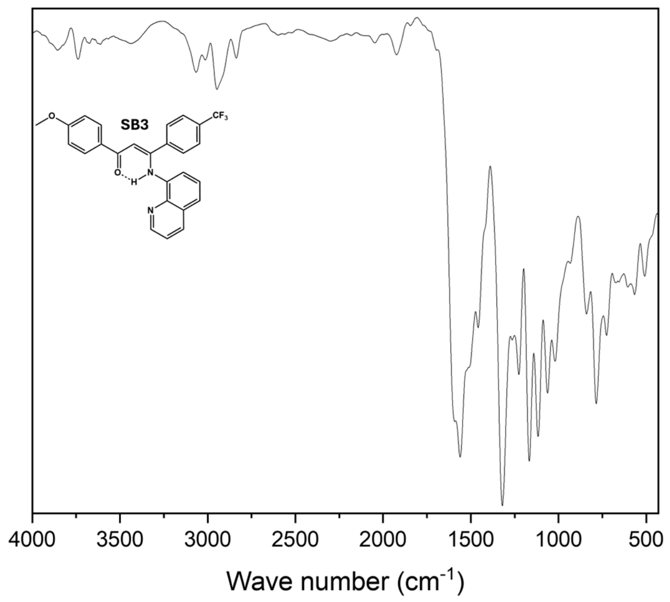

Figure S6. Solid-state FT-IR (KBr) of compound **SB3**.

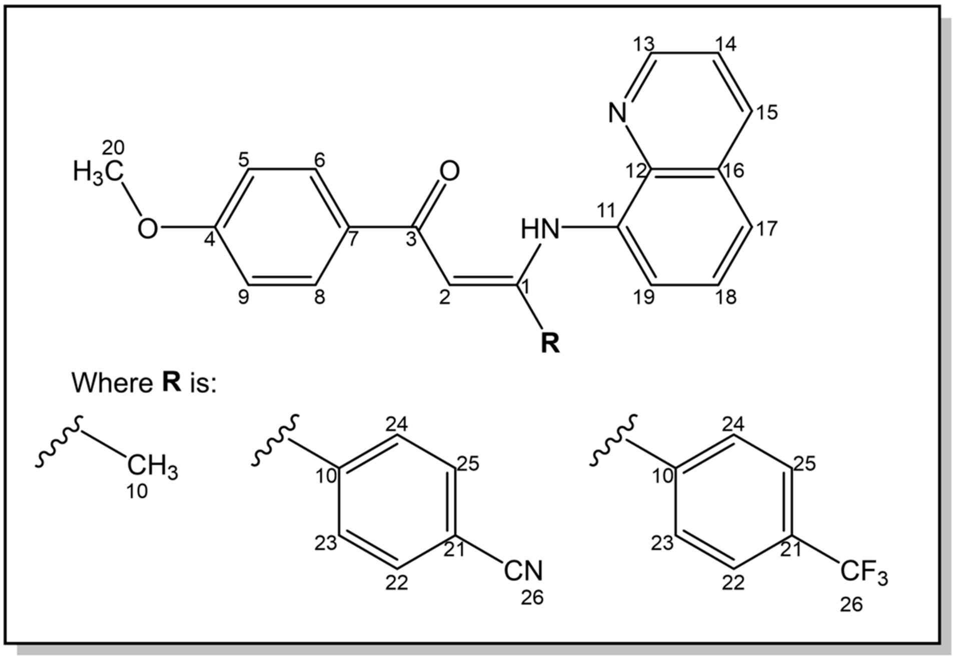

Chart S1. Molecular structure with atomic numbering of **SB1-SB3**.

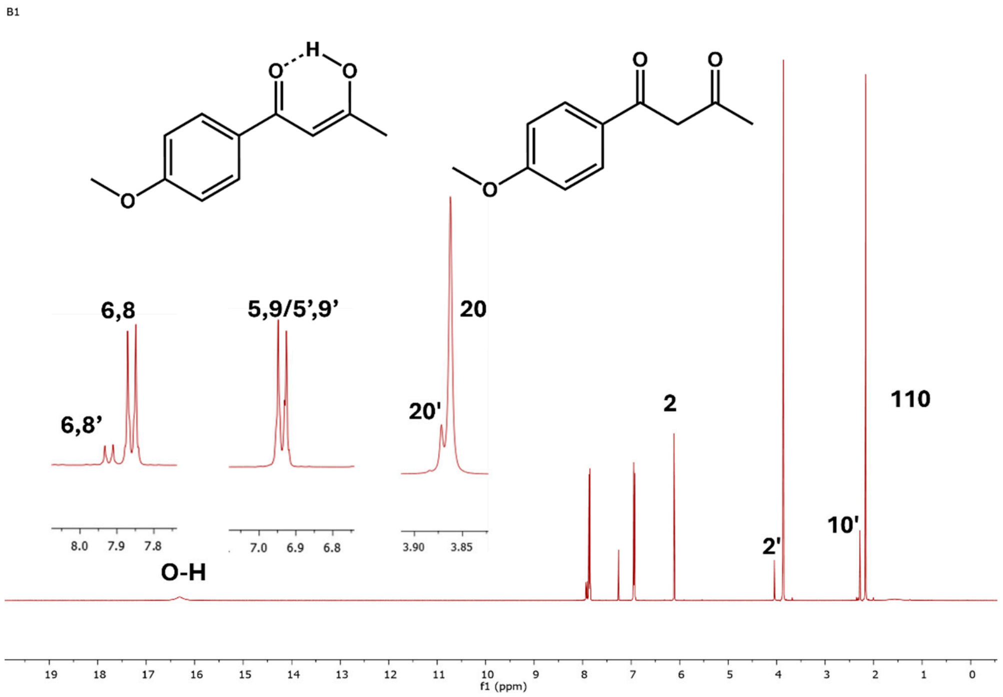

Figure S7. ^{1}H NMR spectrum of compound β1, tautomeric mixture, collected in $CDCl_3$ (400 MHz) at 25 °C.

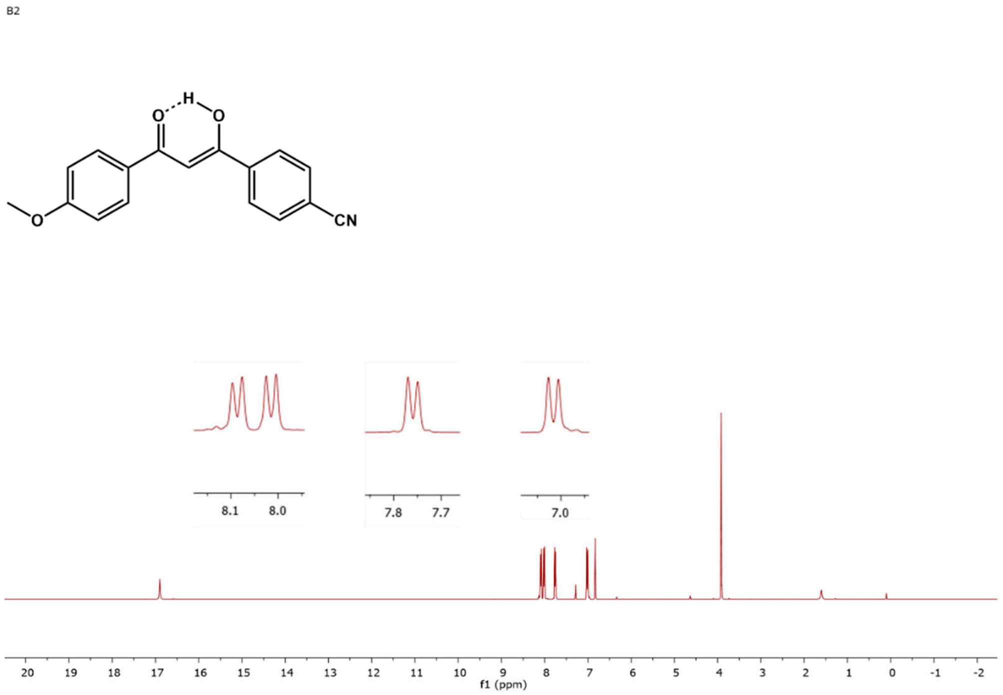

Figure S8. ^{1}H NMR spectrum of diketone B2, collected in $CDCl_3$ (400 MHz) at 25 °C.

B3

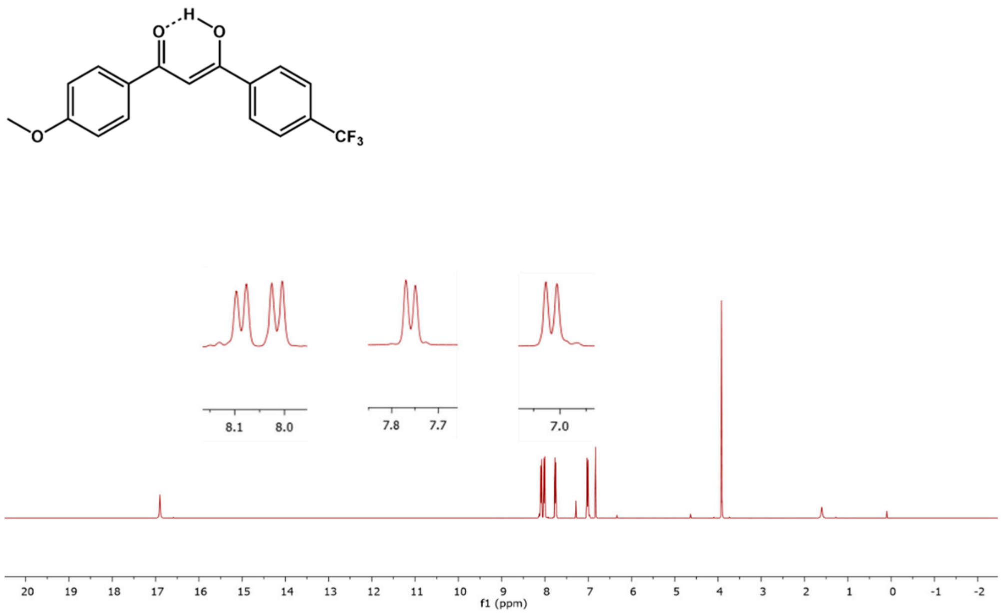

Figure S9. ^{1}H NMR spectrum of compound β3, collected in $CDCl_3$ (400 MHz) at 25 °C.

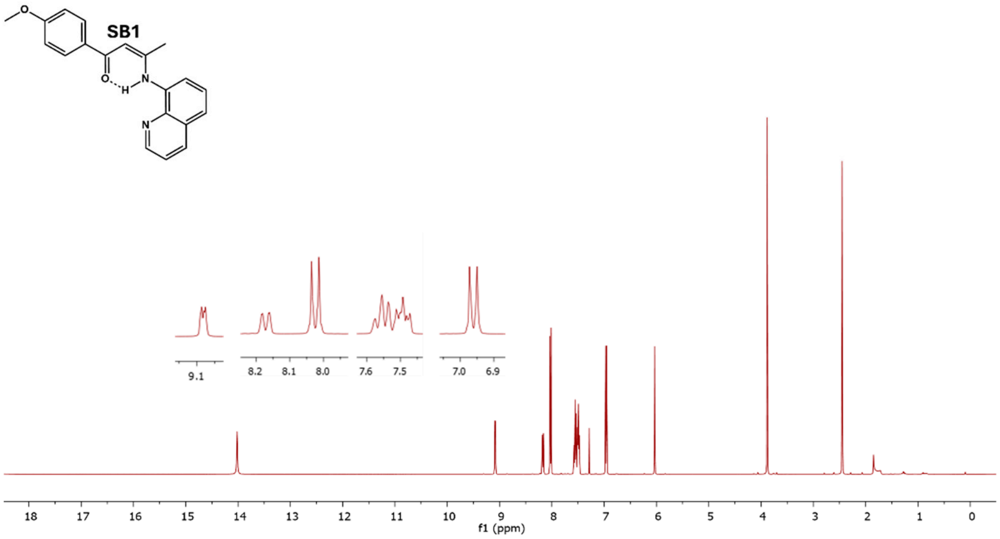

Figure S10. ^{1}H NMR spectrum of compound **SB1**, collected in $CDCl_3$ (400 MHz) at 25 °C.

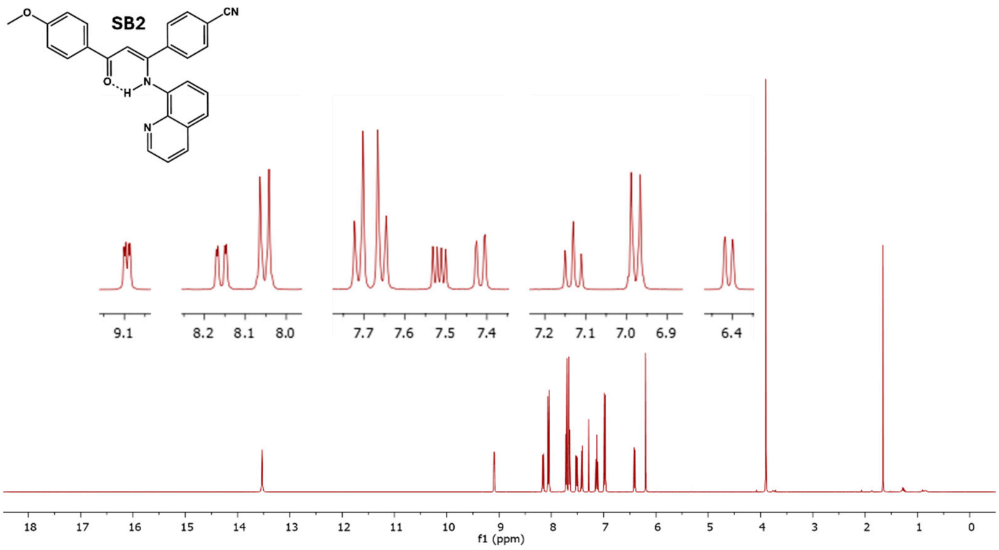

Figure S11. ^{1}H NMR spectrum of compound **SB2**, collected in $CDCl_3$ (400 MHz) at 25 °C.

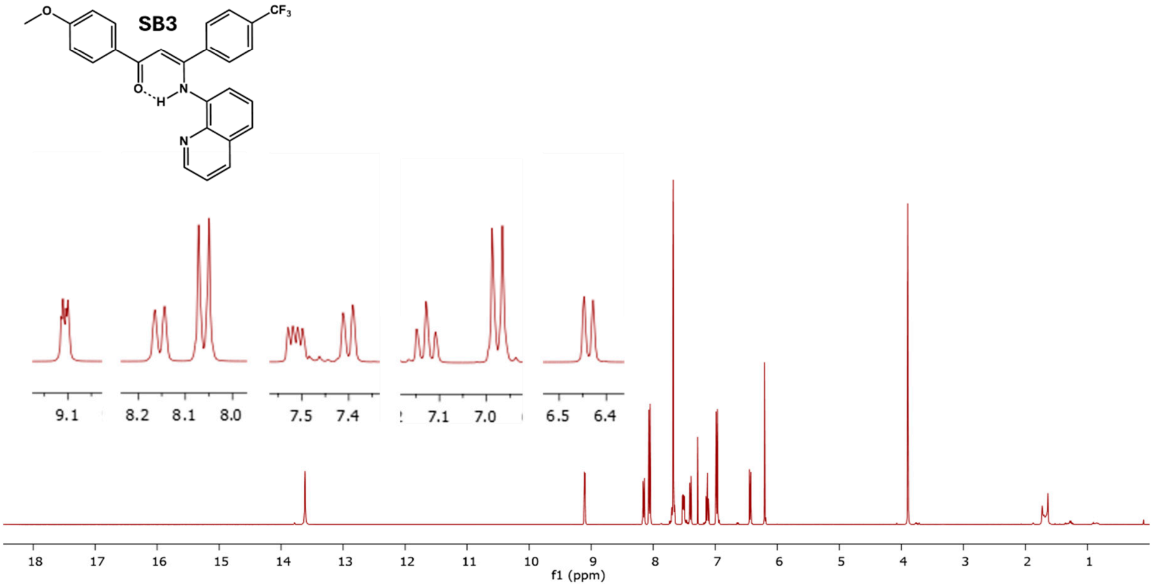

Figure S12. ^{1}H NMR spectrum of compound **SB3**, collected in $CDCl_3$ (400 MHz) at 25 °C.

Table S2. ^{1}H NMR chemical shifts of compounds β1–3 and **SB1- SB3**.[a,b]

**Proton**	**B1**	**B2**	**B3**	**SB1**	**SB2**	**SB3**
O-H	16.31	16.84	16.90	--------	--------	--------
N-H	--------	--------	--------	14.01	13.53	13.58
H2	6.11	6.82	6.82	6.03	6.20	6.21
H6, H8	7.86	8.02	8.02	8.02	8.05	8.06
H5, H9	6.94	7.02	7.02	6.96	6.98	6.98
H10	2.17	--------	--------	2.45	--------	--------
H13	--------	--------	--------	9.08	9.09	9.11
H14	--------	--------	--------	7.52	7.52	7.51
H15	--------	--------	--------	8.18	8.16	8.13
H17, H18, H19	--------	--------	--------	7.52	7.42, 7.13, 6.41	7.40, 7.13, 6.44
H20	3.86	3.93	4.06	3.88	3.88	3.87
H22, H25	--------	8.08	8.09	--------	7.71	7.68
H23, H24	--------	7.80	7.76	--------	7.66	7.68

[a]Spectra recorded in $CDCl_3$ (400 MHz) at 25 °C. [b]For atom labeling see Chart S1 above.

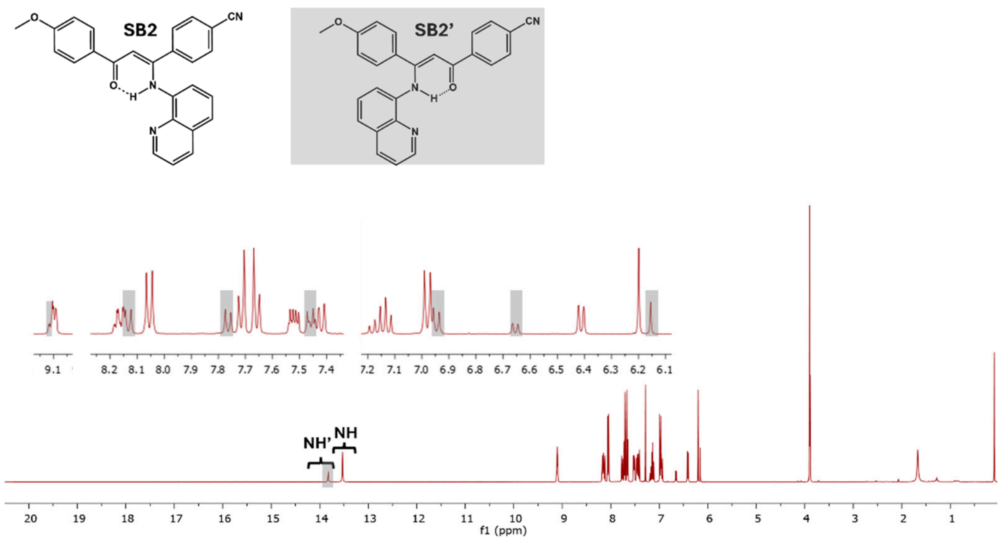

Figure S13. ^{1}H NMR of regioisomeric mixture of compounds **SB2** and **SB2'**, collected in $CDCl_3$ (400 MHz) at 25 °C.

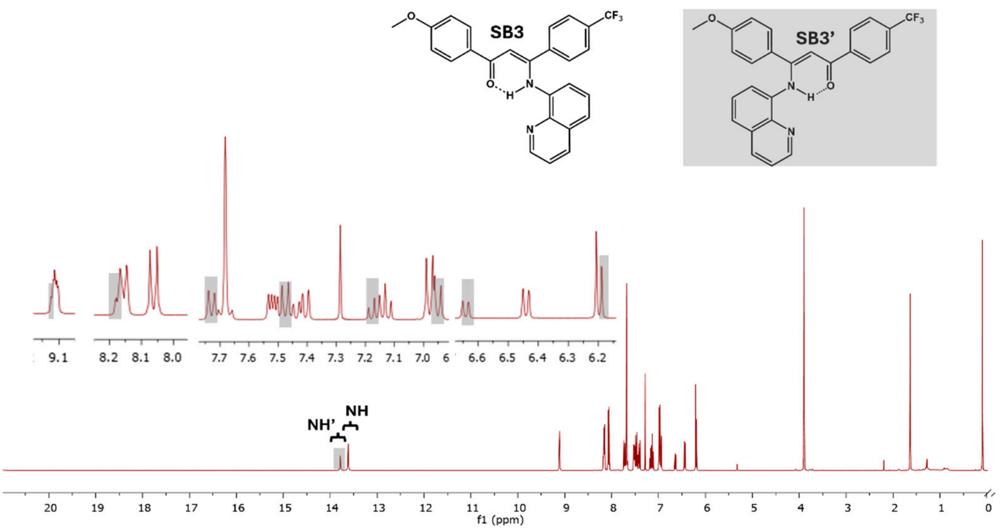

Figure S14. ^{1}H NMR of regioisomeric mixture of compounds **SB3** and **SB3'**, collected in $CDCl_3$ (400 MHz) at 25 °C.

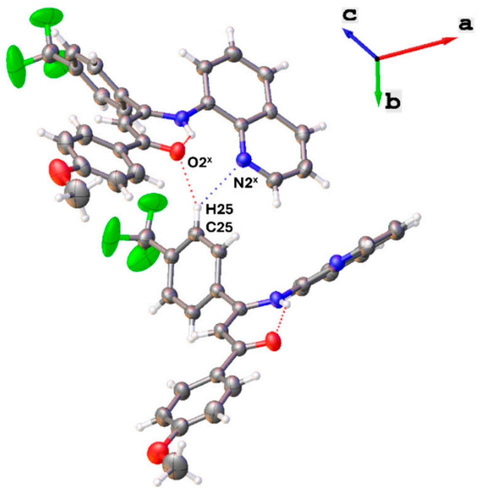

Figure S15. Intermolecular hydrogen bonds C25-H25···O2^{ix} and C25-H25···N2^{ix} in **SB3.**

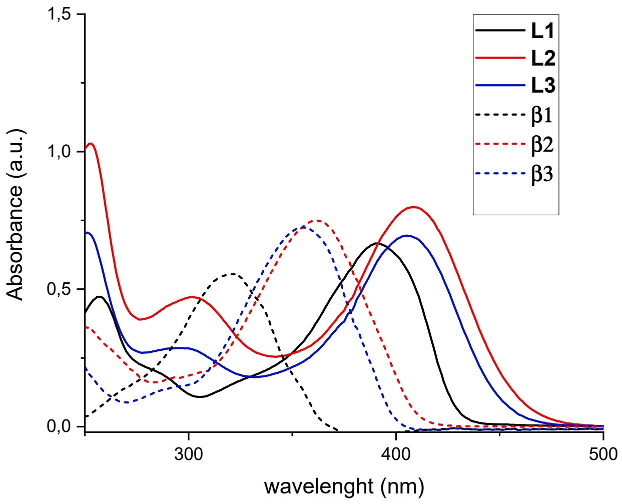

Figure S16. Overplot UV-visible spectra of precursor **β1-3**, and their tridentate ONN **SB1-SB3** recorded in DCM solutions at 20 °C.
